# The effects of binge drinking on attention in young adults

**DOI:** 10.3389/fpsyg.2023.1147621

**Published:** 2023-11-28

**Authors:** Lauren A. Monds, Matthew R. Singleton, Alex M. T. Russell

**Affiliations:** ^1^Specialty of Addiction Medicine, Central Clinical School, University of Sydney, Sydney, NSW, Australia; ^2^Drug and Alcohol Services, Northern Sydney Local Health District, NSW Health, Sydney, NSW, Australia; ^3^Experimental Gambling Research Laboratory, School of Health, Medical and Applied Sciences, CQUniversity, Sydney, NSW, Australia

**Keywords:** attention, binge drinking, cognitive impairment, young adults, alcohol

## Abstract

**Introduction:**

Alcohol binge drinking is highly prevalent among young adults. While research has established the neurotoxic effects of general alcohol consumption, binge drinking presents unique deleterious effects on the brain through the acute intoxication and withdrawal cycle. The detrimental impacts of binge drinking have been reported across a broad range of cognitive abilities in young adults, however, the research regarding its relationship to attention is mixed. This study investigates the relationship between binge drinking and attention performance in young adults. Moreover, there is evidence to suggest that males and females are uniquely impacted by the neurotoxic effects of binge drinking, so the present study tests the moderating role of sex, as well as the influence of earlier age of binge drinking onset.

**Methods:**

One-hundred and five university students were recruited for the study. After collecting socio-demographic, and alcohol use information, participants completed four cognitive tasks designed to measure the three attention networks according to the Attention Network Theory; alerting, orienting, and executive control. Linear hierarchical regressions were used to predict performance with binge drinking score, sex and age of first binge drinking session as predictors.

**Results:**

Binge drinking, sex, and age of first binge drinking session did not predict attention impairment, nor did sex moderate the relationship, at least in the selected cognitive tasks. The tasks used to measure attention did not relate in the expected manner.

**Discussion:**

While there were no differences in attention performance between those who binge drink and controls in this study, the relationship between binge drinking and attention impairments in young adults may be more nuanced and future research directions are suggested. Theoretical and practical implications of these findings are discussed.

## Introduction

Binge drinking is a worldwide issue with 18.2% of the global population (15+ years old) having at least monthly binge drinking occasions (World Health Organization, 2019). Unsafe drinking levels are especially pronounced in young adult populations (18–24 years old). In 2017–2018, 61% of young adults engaged in binge drinking (Australian Bureau of Statistics, 2018). While the specific definition of binge drinking changes across countries, globally it is recognized as drinking large quantities of alcohol in a single session, resulting in intoxication. Although campaigns to change binge drinking attitudes among young adults have achieved some success (Young et al., 2018), given the continued prevalence of binge drinking, further research is warranted.

Binge drinking has especially deleterious effects on the brain relative to regular low-moderate dose alcohol consumption, due to the repeated alternations between acute intoxication followed by withdrawal and abstinence periods (Maurage et al., 2012). In addition, during adolescence, the brain undergoes maturation, and is thus a vulnerable time for insults to the brain, like in heavy alcohol use (Zeigler et al., 2005). This time period in brain development may also be a window which promotes increased binge drinking through insensitivity to the natural aversions of alcohol consumption, and a heightened sensitivity to the rewarding elements of alcohol (Hargreaves et al., 2009). Correspondingly, brain impairments in youth binge drinkers, particularly in higher-order cognition, have been found (Stephens and Duka, 2008; Crego et al., 2009), accompanied by deficits to visual processing and attentional allocation (Maurage et al., 2012), and neurological impairments to memory, language skills, and attention (Hermens et al., 2013).

Attention is the broad term for a set of cognitive processes responsible for the selection and processing of stimuli from one’s perceptual field, while minimizing interference from non-relevant stimuli. Although the many sub-components of attention are related, they can be differentially affected by neurotoxicity. Therefore, the need to study attention in young adults who binge drink relates to an increased vulnerability to parts of the brain responsible for attention, and to clinical implications of early intervention and prevention of progression from binge drinking to AUD (Bonomo et al., 2004).

However, findings on attentional deficits in young adults who binge drink are mixed. Carbia et al. (2018) concluded from their systematic review that binge drinking had a minimal effect on attention. However, in other research, females who binge drink experienced detriments to attention compared to female non-binge drinkers (Squeglia et al., 2012) and in attentional shifting, maintenance, and flexibility (Scaife and Duka, 2009). These mixed findings highlight methodological and theoretical limitations in the research. For example, binge drinking history is often neglected in sampling which ignores the progressive nature of early alcohol-related impairment (Sachdeva et al., 2016). The neurotoxic consequences of binge drinking occur relatively quickly (McQueeny et al., 2009), and age of onset has been found to be a contributing factor to frontal lobe impairment (Townshend and Duka, 2005). Therefore, assessing the relationship between binge drinking age of onset and attentional impairment could provide a predictive tool, examining the extent of impairment over time which would have clinical and public health utility.

A further methodological concern is the fact that different attention processes are measured with a variety of tasks, potentially producing inconsistent results which render the comparison of current studies difficult, and the conclusions about the impact of binge drinking on attention, tentative. Establishing attention in a framework would provide specificity of what is being measured, and guide task selection for studies, allowing for more precision in locating deficits and informative conclusions about what underpins alterations in attentional processes due to binge drinking.

The Attention Network Task (ANT—Fan et al., 2002) is a behavioral measure that assesses the three attention networks (alerting, orienting, and executive control) that underpin the structure of attention according to Attention Network Theory of Posner and Peterson (1990) (Petersen and Posner, 2012). It also provides evidence for the neural dissociations between the three networks (fMRI—Fan et al., 2005). *Alerting* produces and maintains vigilance and performance during a task. *Orienting* is the ability to shift attention to a location in the environment, while *executive control* is the ability to resolve conflict between competing stimuli by focusing on the task-relevant stimulus. The ANT has been widely validated in many populations (Fan et al., 2002; Mullane et al., 2011; Ishigami et al., 2013), including alcohol dependence (Maurage et al., 2014). Lannoy et al. (2017) examined the effect of binge drinking on young adults’ attention using the ANT, finding impairments to the alerting, and executive control networks. There was a ceiling effect in the accuracy measures of the ANT (impairments were identified by participant reaction times) which the researchers attributed to task simplicity, so the full extent of the impairments may not have been captured. Therefore, further research using the ANT and complimentary tasks to capture impairment that may have been missed is warranted. By including other measures of attention alongside the ANT, this study can harmonize the attention literature by seeing how these tests relate to each other, and which measures to use in future research.

Another concern is that most studies group males and females together. Research suggests that the rates and patterns of brain maturation differ according to sex (Lenroot et al., 2007). Moreover, compared to males, it appears that females have an increased sensitivity to the neurotoxic effects of alcohol, showing greater deleterious effects on brain maturation (Squeglia et al., 2012) due to binge drinking, and a faster progression of brain atrophy and impairment throughout alcohol dependence (Mann et al., 2005; Alfonso-Loeches et al., 2013). These sex variations in patterns of brain maturation and physical response of the brain to alcohol correspond to early findings of differences in impairment to attention.

Females were impaired relative to matched controls more than males on sustained attention (Townshend and Duka, 2005), and only females showed impairments on a task measuring attentional maintenance, shifting, and flexibility (Scaife and Duka, 2009). However, the task used here also measures executive function, rule acquisition, and cognitive flexibility which makes concluding about impairments related strictly to attention unrealistic, and further research on sex differences focused on just attention is needed.

### The current study

This study aims to provide conceptual replication of previous research regarding the impact of alcohol on the attentional networks (Lannoy et al., 2017). Due to the ceiling effect on accuracy measures in the ANT in previous research, additional tasks designed to measure each attention network were administered to ensure the full extent of impairment is captured and to investigate their relationship to the ANT. Moreover, it will extend the literature by assessing the moderating influence of sex. Finally, since much of the literature ignores participant history of binge drinking in its testing, the present study will assess the effect of binge drinking history on attentional impairment. The hypotheses are 3-fold:

1. Binge drinking will predict impairment to the alerting and executive control attentional networks.
2. That sex will moderate the relationship between binge drinking and attentional impairment so that females who binge drink will exhibit greater impairment on the tasks compared to their male counterparts.
3. That earlier age of onset of binge drinking will predict more significant impairment to the alerting and executive control networks.

## Materials and methods

### Design

This study adopted a cross-sectional design. There are 15 dependent variables across four tasks, counterbalanced, designed to measure three independent networks of attention: alerting, orienting, and executive control. Hierarchical multiple linear regression was used to test the unique effect of each of the predictor variables of interest (binge drinking score, sex, binge drinking history, and a binge drinking score by sex moderating variable) on these attention networks.

### Participants

Students (*N* = 105; 59% female) were recruited from the University of Sydney and received course credit for participation. Participants were instructed to abstain from the use of sedating medication (e.g., sleeping tablets, antihistamine) for at least 48 h before testing, and alcohol for at least 24 h before testing to rule out acute effects of these substances on cognition. Participants were screened prior to testing to ensure alcohol abstinence compliance. The age ranged from 17 to 43 years old (*M* = 20.03, *SD* = 3.16), with only five participants above the age of 24 years old, who were left in the analysis in consideration of statistical power.

### Measures

#### Questionnaires

##### Socio-demographics

Age, sex, highest level of educational attainment, personal and/or direct familial alcohol dependence (yes/no response), current medication use, and age of first binge drinking session (defined as more than four standard drinks in one sitting) were collected.

##### Alcohol use disorder identification test

The alcohol use disorder identification test (AUDIT) assesses global alcohol intake and exhibits strong psychometric properties (see Reinert and Allen, 2007, for a review). Reliability for the current study is Cronbach’s α = 0.764 (Babor et al., 2001).

##### Alcohol use questionnaire

The final three items (10: average drinks per hour, 11: number of times drunk in the previous 6 months, and 12: percentage of times drunk when drinking), which distinguish between fast and slow drinkers, only have been used to create a *binge drinking score* for each participant (Mehrabian and Russell, 1978). The score is calculated using the formula: [(4 × Item 10) + Item 11 + (0.2 × Item 12)] (Townshend and Duka, 2002). The distribution of raw binge drinking scores has a median of 15, a mean of 18.92 (*SD* = 15.34), and is right skewed, with a maximum observed score of 63. Reliability for the current study is low, Cronbach’s α = 0.338, but as discussed below, this is likely not a concern for this particular scale.

##### Drug use disorder identification test

The drug use disorder identification test (DUDIT) is scored in a similar way to the AUDIT and shows strong reliability and validity in clinical and non-clinical samples (Berman et al., 2005; Durbeej et al., 2010). It was included as a control as drug use also impacts upon attention (Lundqvist, 2005). Reliability in the current study is Cronbach’s α = 0.867 (Berman et al., 2005).^1^

### Cognitive tasks

#### The attention network test

Participants had to determine as quickly and as accurately as possible whether a central arrow, the target, points left or right, which is presented either above or below a central fixation point on the screen (Fan et al., 2002). They respond by pressing the corresponding button on the keyboard (“E” for left, “I” for right). Each target is preceded by either no cue, a central cue (asterisk over the central fixation cross), a double cue (an asterisk above *and* below central fixation cross), or a spatial cue (an asterisk either above *or* below the fixation cross, indicating the location of the upcoming target). Each central target arrow is either presented with no flankers, or two flanking arrows either side in the same direction as the target (congruent trial) or opposite direction (incongruent trial). Each trial is structured: a central fixation cross (random duration 400–1,600 ms), then a cue (100 ms), back to central fixation cross (400 ms), then a target and its flankers (until participant responds with a maximum 1,700 ms for no response), and back to central fixation cross (3,500 ms minus the sum of first fixation duration and target reaction time). One trial last 4 s. There are 288 trials, broken up in to three 96-trial blocks, with a 24-trial practice block to begin with. Trials are presented in random order, with 48 potential trials, two shown in each block.

Reaction time (RT; in ms) and accuracy (AS; percentage of correct responses) were recorded for each trial. From these, mean RT and AS are derived for each attention network. *The alerting effect* is calculated by subtracting the mean score of the double cue trials from the mean score of the no cue trials. *The orienting effect* is calculated by subtracting the mean score for the spatial cue trials from the mean score of the central cue trials. *The executive control effect* is calculated by subtracting the mean score for the congruent flanker trials from the mean score of the incongruent flanker trials. For the alerting and orienting effects, greater RT (and lower AS) scores reflect greater efficiency, and for executive control, lower RT and AS scores reflect greater efficiency. The ANT shows adequate to good test–retest reliability in development (Fan et al., 2002), split-half reliability, stability and robustness of attention networks in healthy university participants (Ishigami and Klein, 2010).

#### Color word Stroop task

The Stroop task was included as a secondary measure of the executive control network (Stroop, 1935). Participants are presented with color words (eg. yellow or blue) on screen and asked to determine the color the word is written in (the color of the font, not the meaning of the text) as quickly and accurately as possible by responding on the keyboard. There are four color words (red, green, blue, and black) that can be presented in three ways: color word and font color correspond (congruent trial), color word and font color do not correspond (incongruent trial), or a control trial (colored rectangle where participants indicate the color of the rectangle). Each of the 12 combinations are presented randomly seven times each making 84 trials total. The stimuli remained on screen until there was a response with a 200 ms inter-trial interval. Reaction time and accuracy of each trial is recorded, and the interference effect, reflecting executive control, is calculated across both measures. For reaction time interference, the mean RT of correct congruent trials is subtracted from the mean RT of correct incongruent trials, with higher scores indicating less executive control efficiency. For accuracy interference, the amount of correct incongruent trials is subtracted from the amount of correct congruent trials, with higher scores indicating less executive control efficiency.

#### Rapid visual information processing

The RVIP was included as a secondary measure of the alerting network (Wesnes and Warburton, 1984). The participant is presented with a series of individual numbers (1–9) on the screen in a random order and must hit the response key (spacebar) as soon as they detect a series of three consecutive odd or even numbers (for example 2-4-6, or 5-7-9). The presentation speed is 100 numbers per minute, with a total of 1,200 numbers presented, and 96 target sequences. There was a practice block of 20 numbers with two target sequences to begin with. Target sequences are separated by a minimum of 5 and a maximum of 33 numbers. The response is scored as a hit if it occurs within 1,500 ms of the onset of the final number in the sequence, otherwise it is counted as a false alarm. The variables of interest are responses made in error (false alarms), and probability of a correct response.

#### Sustained attention to response task

The sustained attention to response task (SART) was included as another measure of alertness (Robertson et al., 1997). It is a type of Go/NoGo task where the participant is presented with a series of individual numbers (1–9) in the middle of the screen in varying sizes. The number appear, and then disappears after a short while and is replaced by a mask, a circle with an “X” in the middle. Participants are required to respond (hit the spacebar on the keyboard) if any number other than “3” appears (Go), and to withhold the response if a “3” appears (NoGo). Each number is randomly presented 25 times each, making a total of 225 trials. The number appears for 250 ms, then the mask for 900 ms. The variables of interest are commission errors (error in NoGo trial), omission errors (error in Go trial), total errors (commission + omission errors), mean reaction time of valid and correct Go trials, and reaction time variability. The SART appears to be a reliable measure of sustained attention and shows good ecological validity (Smilek et al., 2010).

### Procedure

Participants were provided with a Dell desktop computer with Windows 10 operating system in a private cubicle. After reading a Participant Information Statement and providing informed consent, participants filled out the socio-demographic, psychological, and drug/alcohol use questionnaires hosted on Qualtrics and were then automatically forwarded to the cognitive tasks hosted on Inquisit which were counterbalanced. Instructions for each task were provided as the participant reached each task. After the completion of all the tasks, the participant was provided with a Participant Debrief Statement. The total testing time was 1 h. The current study was approved by the Human Research Ethics Committee at the University of Sydney (project number: 2019/432).

### Statistical analysis

The variables of interest are presented in Table 1 according to the attention network they are measuring.

**Table 1:** Variables of interest according to attention network.

Attention network	Task	Dependent variable
Alerting	Attention network task	Alerting reaction time (RT)
		Alerting accuracy score (AS)
	Rapid visual information processing (RVIP)	Errors (false alarms)
		Probability of correct response
	Sustained attention to response task (SART)	Omission errors
		Commission errors
		Total errors
		Reaction time (RT)
		Reaction time variability
Orienting	Attention network task	Orienting reaction time (RT)
		Orienting accuracy score (AS)
Executive control	Attention network task	Executive control reaction time (RT)
		Executive control accuracy score (AS)
	Color Word Stroop Task	Reaction time (RT)
		Accuracy score (AS)

All data analysis was conducted using SPSS statistical analysis software (IBM Corp, 2016). Critical alpha was 0.05. To increase power of the analysis, education, and age were removed from analysis due to their minimal relation to the dependent variables.^2^ Independent *t*-tests (two-tailed) were used to test differences between males and females on the sample characteristics and independent variables. Correlation analyses of the DVs were used to test convergence and divergence of the four tasks. As instrument order was counterbalanced, potential order effects were examined and none were found.

Hierarchical multiple linear regressions were conducted after checking for multicollinearity to test the three hypotheses. Fifteen regression models were executed, one for each dependent variable, with global alcohol intake (GAI), drug use (DU), and direct familial dependence on alcohol (F. Dep) entered in Block 1. Block 2 contained the predictors of the continuous mean-centered binge drinking score, dichotomous sex variable, and dummy-coded age of first binge drinking session variable, and the moderating cross-product variable of sex and mean-centered binge drinking score was entered in Block 3. Results from block 3 are presented. Due to the relationships between some of the dependent variables, alpha was set at 0.003 (0.05/15 dependent variables) for the regression analyses.

## Results

### Preliminary analysis

#### Sample characteristics

The sample demographic, and measures of drug and alcohol use are presented in Table 2, split by gender. There were no significant differences between the groups in age and level of education. Males had a significantly higher global alcohol intake and binge drinking score compared to females.

**Table 2:** Sample characteristics by gender (*N* = 105).

Measure	Male (*n* = 43)	Female (*n* = 62)	*t*-test
Age, mean (SD)	19.88 (2.27)	20.13 (3.67)	*t*(103) = −0.39, *p* = 0.70
Highest level of education, median	Higher school certificate (year 12)	Higher school certificate (year 12)	*t*(101) = 0.39, *p* = 0.70
Age of first binge drinking session, mean (SD)	16 (1.56)	17 (1.48)	*t*(76) = −0.78, *p* = 0.39
AUDIT, mean (SD)	8.63 (5.86)	6.34 (5.22)	*t*(103) = 2.10, *p* = 0.038*
DUDIT, mean (SD)	3.12 (6.00)	1.98 (3.79)	*t*(103) = 1.19, *p* = 0.24
Binge drinking score, mean (SD)	22.61 (15.87)	16.35 (14.55)	*t*(103) = 2.09, *p* = 0.039*

AUDIT, Alcohol use disorder identification test; DUDIT, Drug use disorder identification test.

#### Attention task means

For the purposes of replication (Lannoy et al., 2017), participants with a binge drinking score of ≤12 were classified as control participants (CP, *n* = 42; 44.7% of those who completed the binge drinking measure), and those with a binge drinking score of ≥16 were classified as participants who binge drink (BD, *n* = 52, 55.3%). Mean (SD) AUDIT scores for BD was 10.92 (SD = 4.99, median = 3), which was significantly higher than CP (*M* = 3.70, *SD* = 3.39, median = 10), *Welch t*(89.57) = 8.66, *p* < 0.001, *d* = 1.70.

**Figure 1:**
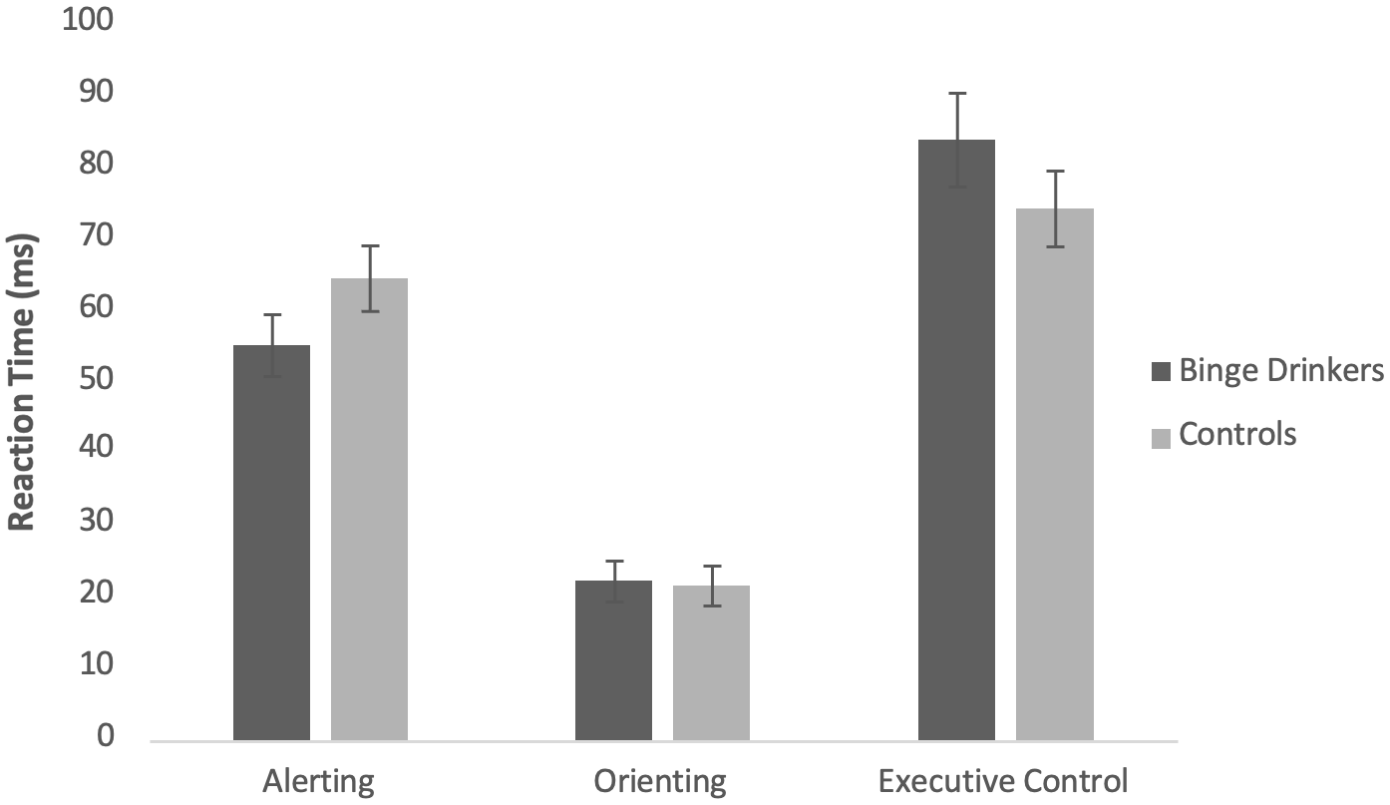
Efficiency of each attention network as a function of reaction time (in ms) among those who binge drink and control participants. Bars represent the mean score, and whiskers are the standard error. Measures are ANT alerting, ANT orienting, and ANT executive reaction times, respectively. For alerting and orienting, greater scores equal greater efficiency, and for executive control, lowers scores equal greater efficiency. There are no significant differences between groups.

Contrary to hypotheses, independent samples *t*-tests found no significant differences between the groups, and the means for each dependent variable and inferential test results are presented in Table 3. The reaction time means in the ANT for participants are presented in Figure 1. Although there were no significant differences between groups, numerically the means fall in the expected direction. That is, control participants had higher scores for the alerting network, indicating greater efficiency, and lower scores for executive control, also indicating greater efficiency compared to participants who binge drink.

**Table 3:** Dependent variable means (SD) as a function of binge drinking status.

Attention network	Dependent variable	Binge drinking status		*t*-test
		CP	BD	
Alerting	ANT alerting RT	64.20 (29.39)	54.96 (29.50)	*t*(88) = 1.48, *p* = 0.143
	ANT alerting AS	−0.07 (3.16)	0.42 (3.98)	*t*(87) = −0.63, *p* = 0.528
	RVIP errors	40.41 (58.52)	34.48 (41.11)	*t*(84) = 0.55, *p* = 0.586
	RVIP prob. correct	47.20 (14.89)	41.19 (15.31)	*t*(84) = 1.83, *p* = 0.072
	SART omission	1.48 (1.54)	1.84 (2.27)	*t*(87) = −0.86, *p* = 0.390
	SART commission	52.95 (22.50)	52.42 (27.12)	*t*(89) = 0.10, *p* = 0.920
	SART total errors	54.43 (23.30)	54.75 (28.93)	*t*(89) = −0.06, *p* = 0.954
	SART RT	341.90 (65.51)	365.90 (87.02)	*t*(89) = −1.46, *p* = 0.148
	SART RT variability	0.26 (0.08)	0.27 (0.09)	*t*(88) = −0.20, *p* = 0.845
Orienting	ANT orienting RT	21.56 (17.61)	22.28 (19.37)	*t*(87) = −0.18, *p* = 0.857
	ANT orienting AS	−0.17 (2.22)	−0.90 (2.72)	*t*(85) = 1.35, *p* = 0.181
Executive control	ANT executive RT	73.99 (34.12)	83.44 (44.50)	*t*(88) = −1.11, *p* = 0.269
	ANT executive AS	12.90 (19.65)	13.00 (20.37)	*t*(85) = −0.02, *p* = 0.982
	Stroop RT	188.19 (203.21)	145.75 (167.39)	*t*(87) = 1.07, *p* = 0.286
	Stroop AS	7.40 (7.92)	7.61 (8.31)	*t*(87) = −0.12, *p* = 0.452

ANT, Attention network task; RVIP, Rapid visual information processing; SART, Sustained attention to response task; RT, Reaction time; and AS, Accuracy score.

### Convergence of attention tasks

A bivariate correlation analyses was employed to examine the relationship between all the measures to see if they related in the expected manner, i.e., the tests measuring alerting should be somewhat related, but unrelated to tests measuring orienting or executive control. The correlations are presented in Table 4. Of the dependent variables used to measure alerting, ANT alerting RT was significantly positively related to SART omission errors, commission errors, and total errors, and SART RT variability, indicating that as participants’ alerting efficiency increased as a function of ANT RT, alerting efficiency decreased as measured by SART errors. The probability of correct responses in the RVIP was significantly negatively related to SART omission errors, and SART RT variability, indicating that as alerting efficiency increased according to the RVIP, so did alerting efficiency in the SART. SART RT was strongly and negatively correlated with all SART error scores, indicating a trade off as RT decreased, errors increased.

**Table 4:** Bivariate correlation analysis of dependent variables.

Attention network	Dependent variable	1	2	3	4	5	6	7	8	9	10	11	12	13	14	15	16
	1 Binge drinking (categorical, ref. = no)	1															
Alerting	2 ANT alerting RT	−0.16	1														
	3 ANT alerting AS	0.07	0.07	1													
	4 RVIP errors	−0.06	−0.05	−0.12	1												
	5 RVIP prob. correct	−0.20	−0.12	−0.05	−0.07	1											
	6 SART omission	0.09	0.40***	−0.03	0.03	−0.26*	1										
	7 SART commission	−0.01	0.28**	−0.15	0.08	−0.14	0.55***	1									
	8 SART total errors	0.01	0.29**	−0.15	0.08	−0.15	0.60***	0.99***	1								
	9 SART RT	0.15	−0.05	0.13	0.06	−0.05	−0.29**	−0.78***	−0.77***	1							
	10 SART RT variability	0.02	0.37***	−0.16	0.18	−0.26*	0.47***	0.39***	0.41***	−0.05	1						
Orienting	11 ANT orienting RT	0.02	0.07	0.02	−0.03	0.09	0.02	−0.04	−0.02	0.04	−0.09	1					
	12 ANT orienting AS	−0.15	0.00	−0.15	−0.04	−0.04	−0.15	0.01	0.00	−0.01	0.11	0.00	1				
Executive control	13 ANT executive RT	0.12	−0.08	0.06	0.11	−0.08	0.08	0.00	0.03	0.03	0.17	−0.12	−0.01	1			
	14 ANT executive AS	0.00	0.21*	−0.15	−0.02	−0.13	0.26*	0.16	0.17	−0.13	0.17	−0.20	0.08	−0.05	1		
	15 Stroop RT	−0.12	0.03	0.08	0.12	−0.06	−0.03	−0.01	0.00	0.07	0.04	0.19	−0.11	0.00	−0.06	1	
	16 Stroop AS	0.01	0.13	0.07	0.03	0.03	0.19	0.12	0.15	−0.05	0.13	−0.03	−0.05	0.22*	0.15	0.22*	1

ANT, Attention network task; RVIP, Rapid visual information processing; SART, Sustained attention to response task; RT, Reaction time; AS, Accuracy score. ^*^p < 0.05, ^**^p < 0.01, and ^***^p < 0.001 two-tailed.

Of the dependent variables used to measure executive control, ANT executive RT was significantly positively related to Stroop AS, indicating that as participants’ executive control efficiency decreased according to ANT executive RT, it also decreased according to the Stroop AS measure. The ANT executive AS was also positively related to SART omission errors, indicating that as executive control efficiency decreased, so did alerting efficiency according to SART omission scores. Stroop RT and AS were also significantly positively related, so that as executive control efficiency decreased according to Stroop RT, it also decreased according to Stroop AS. All other measures were unrelated. Therefore, the ANT executive AS measure was the only measure to relate to other attention network measures, namely, alerting, through the ANT alerting RT and the SART omission errors.

### Regression analysis

The binge drinking history variable was collapsed from eight to three levels (age of first binge drink session: ≤ 15, 16–17, and ≥ 18 years old) in consideration of statistical power and dummy coded with ≤15 years old as the reference group. Sex was a dichotomous variable with males as the reference group. A Poisson distribution was used for RVIP errors, as it was a count variable, while all other dependent variables were continuous variables, and thus linear models were employed. The same models were conducted without the control variables and results are similar (Table 5).

**Table 5:** Regression results.

Dependent variable	BD (cat)	Sex	BD*Sex	Drug use	Direct familial dependence on alcohol	First binge drinking session age (16–17 vs. <15)	First binge drinking session age (18+ vs. <15)	Constant
ANT alerting RT	7.23 (−15.03, 29.49)	17.63 (−2.65, 37.90)	−10.32 (−36.97, 16.34)	−0.56 (−1.87, 0.75)	0.82 (−17.90, 19.55)	−10.81 (−27.06, 5.44)	0.96 (−15.84, 17.76)	52.40*** (34.64, 70.16)
ANT alerting AS	1.58 (−1.10, 4.26)	0.62 (−1.82, 3.06)	−0.77 (−4.00, 2.47)	−0.12 (−0.28, 0.04)	−2.27 (−4.53, −0.02)	0.77 (−1.22, 2.75)	1.60 (−0.43, 3.64)	−0.74 (−2.88, 1.40)
RVIP errors	−0.25*** (−0.40, −0.11)	0.38*** (0.25, 0.51)	0.03 (−0.14, 0.19)	−0.06*** (−0.07, −0.05)	0.65*** (0.56, 0.74)	0.47*** (0.38, 0.56)	0.13* (0.03, 0.23)	3.30*** (3.19, 3.42)
RVIP prob. correct	−4.31 (−16.59, 7.96)	2.80 (−8.38, 13.98)	−5.88 (−20.39, 8.63)	0.17 (−0.53, 0.88)	−2.67 (−12.85, 7.48)	3.62 (−5.04, 12.29)	−0.61 (−9.82, 8.59)	44.87*** (34.99, 54.74)
SART omission	−0.32 (−1.79, 1.16)	−0.21 (−1.54, 1.13)	1.48 (−0.29, 3.25)	0.07 (−0.01, 0.16)	−0.72 (−1.99, 0.55)	−0.36 (−1.44, 0.72)	0.50 (−0.62, 1.61)	1.53* (0.36, 2.70)
SART commission	−1.82 (−20.99, 17.35)	3.86 (−13.60, 21.33)	9.99 (−12.85, 32.82)	0.19 (−0.94, 1.32)	1.27 (−14.74, 17.28)	−5.96 (−19.87, 7.95)	3.21 (−11.26, 17.68)	49.78*** (34.48, 65.07)
SART total errors	−1.40 (−21.62, 18.82)	3.68 (−14.74, 22.10)	11.09 (−13.00, 35.17)	0.19 (−1.00, 1.38)	1.71 (−15.18, 18.60)	−6.39 (−21.06, 8.28)	3.25 (−12.01, 18.52)	51.40*** (35.26, 67.53)
SART RT	43.46 (−16.76, 103.69)	−0.83 (−55.70, 54.04)	−29.42 (−101.16, 42.32)	−1.21 (−4.75, 2.34)	2.09 (−48.22, 52.40)	−7.95 (−51.65, 35.75)	−11.62 (−57.09, 33.85)	346.58*** (298.52, 394.65)
SART RT variability	−0.03 (−0.09, 0.03)	−0.03 (−0.09, 0.02)	0.07 (<0.01, 0.15)	0.00 (−0.001, 0.01)	0.02 (−0.04, 0.07)	−0.05* (−0.09, −0.01)	−0.02 (−0.07, 0.02)	0.29*** (0.25, 0.34)
ANT orienting RT	19.00^**^ (4.97, 33.02)	15.12^*^ (2.24, 28.00)	−20.86^*^ (−37.56, −4.17)	−0.89^*^ (−1.70, −0.09)	−1.38 (−12.87, 10.11)	−3.05 (−13.03, 6.93)	−5.55 (−15.88, 4.78)	12.41^*^ (0.95, 23.87)
ANT orienting AS	−1.47 (−3.34, 0.41)	0.23 (−1.46, 1.93)	1.21 (−1.04, 3.46)	0.05 (−0.07, 0.15)	0.42 (−1.16, 2.00)	0.02 (−1.35, 1.39)	−1.32 (−2.79, 0.14)	−0.14 (−1.63, 1.34)
ANT executive RT	6.82 (−23.76, 37.40)	5.77 (−22.09, 33.62)	−4.71 (−41.32, 31.91)	−0.35 (−2.15, 1.45)	21.48 (−4.25, 47.20)	8.60 (−13.72, 30.93)	2.61 (−20.47, 25.69)	67.60^***^ (43.20, 92.00)
ANT executive AS	−11.24 (−26.61, 4.12)	−7.95 (−21.81, 5.91)	20.78^*^ (2.15, 39.40)	0.06 (−0.84, 0.96)	3.00 (−10.24, 16.24)	−1.03 (−12.41, 10.36)	0.50 (−11.53, 12.54)	18.56^**^ (6.41, 30.71)
Stroop RT	−71.61 (−210.59, 67.37)	−78.57 (−205.76, 48.61)	55.92 (−110.93, 222.77)	−9.42^*^ (−17.60, −1.24)	19.19 (−97.69, 136.08)	18.77 (−82.78, 120.32)	−47.41 (−152.62, 57.79)	260.68^***^ (149.80, 371.56)
Stroop AS	0.17 (−6.19, 6.54)	1.42 (−4.43, 7.26)	−0.40 (−7.97, 7.18)	0.06 (−0.31, 0.42)	−4.74 (−9.95, 0.48)	3.44 (−1.10, 7.97)	2.75 (−1.94, 7.44)	5.58^*^ (0.38, 10.78)

All models are linear models, except RVIP errors, which is a Poisson model. Coefficients are unstandardized coefficients and values in brackets are 95% confidence intervals. ^*^p < 0.05; ^**^p < 0.01; ^***^p < 0.001.

The normality of residuals assumption was violated in the regression analyses related to the SART omission and ANT executive control AS dependent variables and must be considered when interpreting the results. Robust regression models did not change the results.

#### Alerting

A series of hierarchical multiple linear regression were conducted to examine if binge drinking, sex, and age of first binge drinking session predicted performance on the alerting attention network as measured by three tasks across nine dependent variables, and if sex moderated this relationship. None of the models reached statistical significance (all *p*’s > 0.003).

#### Orienting

The ANT orienting RT and AS measures were each regressed on the same three block hierarchical multiple linear regression models. None of the models reached statistical significance (all *p*’s > 0.003).

#### Executive control

The four dependent variables used to measure executive control (ANT executive RT and AS, and Stroop RT and AS) were each regressed on the same three block hierarchical multiple linear regression models. None of the models reached statistical significance (all *p*’s > 0.003).

## Discussion

The present study investigated the relationship between binge drinking, sex and age of first binge drinking session, and attention as conceptualized in Attention Network Theory of Posner and Peterson (1990) and Petersen and Posner (2012). It was hypothesized that binge drinking would predict impairment to the alerting and executive control attention networks, that this impairment would be significantly greater for females who participated in binge drinking compared to males, and that an earlier age of first binge drinking session would also predict greater impairment to these attention networks. Contrary to the hypotheses and across all four tasks, binge drinking and age of first binge drinking session did not appear to predict attentional impairment across any of the three attentional networks, and following that, it appears that sex did not moderate the relationship between binge drinking and attention performance.

### Binge drinking and attention

These findings are in line with some previous research that found no effect of binge drinking on sustained attention (an element of alerting) in adolescence through a longitudinal study (Boelema et al., 2015), and no relation between binge drinking in neuropsychological tests of selective attention, specifically the Stroop Task (Gil-Hernandez and Garcia-Moreno, 2016). The current study appears congruent with the conclusion of review of Carbia et al. (2018) that binge drinking does not seem related to impairments in attention nor does sex moderate the relationship, at least as measured by the ANT, RVIP, SART, and Stroop tasks.

However, while some papers failed to find a relation between binge drinking and attention impairment as measured by similar behavioral neuropsychological tasks, they did find evidence to support prefrontal dysexecutive symptomology and functional or neuro-activational alterations, both related to binge drinking. For example, binge drinking participants performed significantly better than control participants in attention neuropsychological tasks but were significantly impaired on behavior scales measuring everyday prefrontal cortex functionality. Specifically, on a scale designed to measure neuro-behavioral symptoms associated with regions of the brain shared with the executive control attention network (Gil-Hernandez and Garcia-Moreno, 2016).

Caution must be used in reaching conclusions about the relationship between binge drinking and attention impairment in young adults strictly using neuropsychological behavioral measures. They may not be sensitive enough to identify early signs of impairment. Indeed, participants in the current study may have performed at the same level in the ANT and other tasks, but future research involving brain imaging could reveal anomalies for those who binge drink in the activation of the attention network regions, potentially indicating structural and functional changes in attention. This is speculative but warrants further investigation.

In light of this, the current results are divergent to a body of research that provides neuropsychological behavioral evidence for the association between binge drinking and attention impairment (Hartley et al., 2004; Kashfi et al., 2017), and the moderating role of sex in the relationship (Mann et al., 2005; Townshend and Duka, 2005; Scaife and Duka, 2009; Squeglia et al., 2011; Ewing et al., 2014).

Of particular interest are the findings of study of Lannoy et al. (2017) who found, using the ANT that those who binge drank had significantly impaired alerting and executive control networks compared to those who did not. In the present study, when the binge drinking variable was transformed in to a categorical variable, the mean scores, numerically, were in line with the first hypothesis of the current paper and showed the same effect to that seen in the study of Lannoy et al. (2017), although they did not reach significance. However, the regression analyses also appear to suggest that binge drinking does not predict impairment in these attention networks.

Sex was not a predictor of attention performance nor did it moderate the relationship between binge drinking and attention performance. These findings are in line with a systematic review (Carbia et al., 2018) that found minimal differential sex-related neuropsychological effects regarding binge drinking. However, unique spatial working memory deficits in females who binge drink, relative to males, seems to be an established finding and warrants further research into other cognitive faculties (Townshend and Duka, 2005; Scaife and Duka, 2009; Squeglia et al., 2011), but it may be that binge drinking does not uniquely impact the attentional capabilities of males and females. Alternatively, this study did not identify the effect because of being potentially underpowered. Nevertheless, AUD and binge drinking in adolescents has been linked to differential sex effects on prefrontal cortex morphometry which may impact their neurodevelopment uniquely (Medina et al., 2008) and impair attention relative to gender matched controls (Squeglia et al., 2012). Provided the evidence for sex differences in neuroadaptation to alcohol and withdrawal neurotoxicity (Sharrett-Field et al., 2013), and in other cognitive faculties (Scaife and Duka, 2009; Salas-Gomez et al., 2016), future research should be conducted on differential early neurophysiological or functional activation markers of attentional impairments between males and females before no effect is concluded.

### Influence of binge drinking history

The finding that earlier age of first binge drinking session did not predict attention performance was unexpected. In university students, age of binge drinking onset has been found to be a contributing factor to inhibition impairment (Townshend and Duka, 2005), and associated with poorer cognitive flexibility relative to controls (Salas-Gomez et al., 2016). In adolescents who binge drink, earlier age of onset was linked to poorer performance in an attention task throughout a 4-week abstinence period (Winward et al., 2014). These studies suggest that the neurotoxic consequences of binge drinking occur quickly and accumulatively. It may be that the behavioral tests used in the current study were not sensitive enough to identify early, sub-clinical impairments to attention in those who binge drink. For example, one study found that executive function deficits in participants who binge drunk relative to controls only emerged after 6 years of binge drinking maintenance (Gil-Hernandez et al., 2017). Therefore, future research should consider using highly sensitive neuropsychological, neurophysiological, or brain imaging measures when testing younger university samples (in addition to other samples given the potential lack of generalizability of this sample to other population groups) to identify potential sub-clinical, early impairment. Alternatively, behavioral tasks, despite their perceived objectivity, may have lower reliability and potentially lack content validity compared to certain self-report measures of attention, which would be worth exploring in a future study.

### Task selection and theoretical implications

The ANT measures were expected to be independent from each other, and the alerting measures (RVIP, SART, and ANT alerting) to be related to each other, as for the executive control measures (Stroop and ANT executive control). As expected, the ANT measures were all unrelated.

Future research should consider different tasks, if they wish to further measure the alerting network in a complimentary manner to the ANT. For example, a traditionally formatted Go/No-Go task which removes the element of response inhibition.

In addition, the current results may have theoretical implications regarding the selected theory of attention. The attention network theory (Petersen and Posner, 2012), and the ANT, may not be the most suitable means to examine the hypothesized relationships. The point remains that the various forms of attention processes (e.g., selectivity, sustained) arise from the coordination of localized cerebral networks in the brain, the structure of attention (Posner and Rothbart, 2007). It is by examining how binge drinking alters the structure of attention that light may be shed on how the arising attentional processes are influenced, for example, alcohol-related attention biases, and how they may better be rehabilitated. While the Attention Network Theory is one such theory of attention structure, Corbetta and Shulman’s (2002) dual-network theory is another. It stipulates two different neural networks responsible for top-down and bottom-up attentional processing, and identifies regions in the prefrontal cortex that manage their interaction (Fox et al., 2006). Future research could look at how binge drinking impacts the operation of these networks. It is attentional biasing toward increasingly salient alcohol-related cues in the environment that increases the risk of developing AUD from binge drinking (Bonomo et al., 2004), and this may be due to the strengthening and/or deterioration of the neural networks responsible for top-down and bottom-up attentional processing.

The results of the current study must be interpreted considering some limitations. The AUQ scale reliability that the binge drinking score was derived from were low in the current study. However, this is likely due to the nature of the questions rather than concerns about the data. For example, item 10 measures how fast a person drinks when they drink, while item 11 asks how many times they have gotten drunk in the last 6 months. These items were positively correlated (*r* = 0.28, *p* = 0.004), reflecting that some, but not all, people who drink often drink fast. In the long run, drinking both fast and regularly is likely unsustainable, and thus this is reflected in the low reliability. However, this scoring has been used in previous studies (e.g., Townshend and Duka, 2002), minimizing concerns about reliability. Further, the same binge drinking threshold was used for everyone, regardless of sex, in line with scoring of this particular measure. Sex differences in binge drinking thresholds is a potential avenue for future research.

The current experiment ran for 1 h in length and consisted of four attention demanding tests that were measuring elements of attention. While the tasks were counterbalanced to remove the possibility of systematic fatigue, attention toward the end of the experiment would have been drained and could have caused relatively poor task performance within subjects, and potentially encouraged disingenuous responding. However, no order effects were found in terms of performance on any task.

Nevertheless, this study has highlighted the operative and theoretical inconsistencies in the literature, and argued for the need to test attention within a framework concerned with its functional structure. In doing so, a framework of attention can guide task selection for future research that will introduce consistency to what is being measured. In this case, the tasks chosen to compliment the ANT, specifically the alerting tasks, did not relate in the expected manner and raises questions to their suitability in measuring the alerting network, or the suitability of the Attention Network Theory (Petersen and Posner, 2012) in framing attention. Ideally, research can begin to investigate the underlying mechanisms responsible for alterations to the arising attention processes that are part of the trajectory shifting people from a binge drinking pattern of alcohol consumption to alcohol dependency. This will inform the development of new, and refinement of existing clinical rehabilitation efforts. Moreover, the need to use neurophysiological testing, particularly for younger cohorts, to identify early impairments to attention has been established. This research did not receive any specific grant from funding agencies in the public, commercial, or not-for-profit sectors.

## Data availability statement

The raw data supporting the conclusions of this article will be made available by the authors, without undue reservation.

## Ethics statement

The studies involving humans were approved by University of Sydney Human Research Ethics Committee. The studies were conducted in accordance with the local legislation and institutional requirements. The participants provided their written informed consent to participate in this study.

## Author contributions

MS completed this study as a student for his thesis, as part of the requirements of his undergraduate psychology honors degree; he did the majority of the writing and analyses. LM supervised MS for his thesis, helped with study conceptualization, results interpretation, and assisted with write-up. AMT assisted with statistics and interpretation of results. All authors contributed to the article and approved the submitted version.

## Footnotes

^1^The Brief Sensation Seeking Scale (BSSS-8; Hoyle et al., 2002) was included as a correlate measure to binge drinking and the DASS-21 (Lovibond and Lovibond, 1995) was included as a control measure. Because the BSSS-8 is not central to the research question or study outcomes, and the DASS-21 was not related to any DVs, they are not considered further.

^2^Education was only related to ANT alerting AS (−0.248), age related to Stroop RT (−0.242), and ANT alerting RT (−0.249).
